# Modality-level obstacles and initiatives to improve representation in fetal, infant, and toddler neuroimaging research samples

**DOI:** 10.1016/j.dcn.2024.101505

**Published:** 2025-01-03

**Authors:** Emma T. Margolis, Paige M. Nelson, Abigail Fiske, Juliette L.Y. Champaud, Halie A. Olson, María José C. Gomez, Áine T. Dineen, Chiara Bulgarelli, Sonya V. Troller-Renfree, Kirsten A. Donald, Marisa N. Spann, Brittany Howell, Dustin Scheinost, Marta Korom

**Affiliations:** a Department of Psychology, Northeastern University, Boston, MA, USA; b Center for Cognitive and Brain Health, Northeastern University, Boston, MA, USA; c Department of Psychological and Brain Sciences, University of Iowa, Iowa City, IA, USA; d Department of Psychology, Lancaster University, Lancaster, UK; e Department of Neuroscience, Psychology and Pharmacology, University College London, UK; f Centre for the Developing Brain, King’s College London, UK; g McGovern Institute for Brain Research, Massachusetts Institute of Technology, Cambridge, MA, USA; h Research Institute of the McGill University Health Centre, McGill University, Montreal QC, Canada; i Trinity College Institute of Neuroscience, Trinity College Dublin, Dublin 2, Ireland; j School of Psychology, Trinity College Dublin, Dublin 2, Ireland; k Centre for Brain and Cognitive Development, Birkbeck, University of London, London, UK; l Department of Human Development, Teachers College, Columbia University, NY, United States; m Division of Developmental Paediatrics, Department of Paediatrics and Child Health, Red Cross War Memorial Children’s Hospital, University of Cape Town, Cape Town; n The Neuroscience Institute, University of Cape Town, Cape Town, South Africa; o Department of Psychiatry, Vagelos College of Physicians and Surgeons, Columbia University, New York, NY, USA; p Fralin Biomedical Research Institute at VTC, Roanoke, VA, USA; q Department of Human Development and Family Science, Virginia Tech, Blacksburg, VA, USA; r Department of Radiology & Biomedical Imaging, Yale School of Medicine, New Haven, CT, United States; s Department of Biomedical Engineering, Yale University, New Haven, CT, United States; t Department of Statistics & Data Science, Yale University, New Haven, CT, United States; u Child Study Center, Yale School of Medicine, New Haven, CT, United States; v Section on Development and Affective Neuroscience, National Institute of Mental Health, Bethesda, MD, USA

**Keywords:** Fetal, infant, toddler, Neuroimaging, Brain development, Diversity, Inclusive representation, Recruitment

## Abstract

Fetal, infant, and toddler (FIT) neuroimaging researchers study early brain development to gain insights into neurodevelopmental processes and identify early markers of neurobiological vulnerabilities to target for intervention. However, the field has historically excluded people from global majority countries and from marginalized communities in FIT neuroimaging research. Inclusive and representative samples are essential for generalizing findings across neuroimaging modalities, such as magnetic resonance imaging, magnetoencephalography, electroencephalography, functional near-infrared spectroscopy, and cranial ultrasonography. These FIT neuroimaging techniques pose unique and overlapping challenges to equitable representation in research through sampling bias, technical constraints, limited accessibility, and insufficient resources. The present article adds to the conversation around the need to improve inclusivity by highlighting modality-specific historical and current obstacles and ongoing initiatives. We conclude by discussing tangible solutions that transcend individual modalities, ultimately providing recommendations to promote equitable FIT neuroscience.

## 1 Introduction

The field of fetal, infant, and toddler (FIT) neuroimaging faces two major challenges with regards to inclusive and representative samples. First, to date, FIT neuroimaging has predominantly included White populations from countries within the global minority (e.g., those in North America and Europe; Chiao and Cheon, 2010; Duncan and Rae, 2024; Morales et al., 2024; Wedderburn et al., 2020). This selection bias between countries has led to a significant underrepresentation of individuals from countries within the global majority (Alam, 2008, Khan et al., 2022) in FIT neuroimaging research. Second, pervasive sampling bias also exists within global minority countries, where research has historically excluded culturally and politically marginalized populations who are also disproportionately affected by systemic factors that result in socioeconomic and educational inequities, often along racial, ethnic, cultural, and/or religious lines (Henrich et al., 2010). These underrepresented groups include those living in low-resource or rural settings, communities of color, migrant populations, refugees, those with neurodevelopmental conditions, and clinically vulnerable populations, among others. From an intersectional lens, these marginalized identities may overlap in a myriad of ways, producing distinct identities related to additional barriers to representation in research (Crenshaw, 1989, Warner, 2008).

The underrepresentation of marginalized communities in FIT neuroimaging research has profound implications for the field, particularly regarding our understanding of the developing brain. This disparity threatens the validity, reliability, reproducibility, and generalizability of research findings (Green et al., 2022). It also compromises a fundamental objective of FIT neuroimaging, including gaining insights into purported neurodevelopmental markers of vulnerability, which may also disproportionately affect marginalized populations (Dickerson et al., 2023, Dumornay et al., 2023, Graham et al., 2021, Rami et al., 2023). If these neural markers of vulnerability are established only in a subset of the population, other groups may be under-identified as at risk, which could result in misdiagnosis, inadequate interventions, and perpetuation of health disparities (LeWinn et al., 2017). Although the FIT neuroimaging community acknowledges the need to enhance diversity in FIT research (Hendrix and Thomason, 2022), numerous technological, recruitment, financial, and procedural obstacles impede progress. Addressing this critical issue is our ethical responsibility and requires a reflective examination of past and present efforts to engage diverse populations and implement strategies to overcome barriers to equitable developmental neuroscience.

To effectively address these obstacles, it is imperative to identify individual- and environmental-level root causes, which have led to the systemic underrepresentation of groups within the global majority and individuals with marginalized identities from FIT neuroimaging research (Green et al., 2022). For instance, it may be difficult to maintain contact with individuals in lower-resource communities due to decreased flexibility in scheduling around the workday and/or decreased stability of contact methods (e.g., frequent changes in home address or phone numbers) (Schneider and Harknett, 2019, The Children’s Society, 2020). Additional individual-level obstacles include sampling biases stemming from the lack of intentional recruitment of diverse populations, individuals’ reluctance to participate in research due to distrust in the intentions of medical research professionals, as well as barriers to engaging diverse target groups (Green et al., 2022, Habibi et al., 2015, Nielsen et al., 2017, Raphael et al., 2017). The historical medical exploitation and abuse of marginalized groups, especially communities of color, in clinical, research, and public health contexts (Anastas, 2013, Baptiste et al., 2022, Gamble, 1993) has resulted in justified distrust of medical and scientific professionals and technologies (Bazargan et al., 2021, Kennedy et al., 2007), leading to hesitancy to engage in research studies. However, some research suggests that when equitably recruited (Bradford et al., 2024), individuals with marginalized identities are equally likely to participate in research, even when they are aware of historical examples of racist research studies such as the Tuskegee Syphilis Study (Bradford et al., 2024, Fisher and Kalbaugh, 2011, Wendler et al., 2005). Additionally, relatively higher levels of attrition from marginalized groups in longitudinal neuroimaging studies (Ewing et al., 2018) lead to decreased representation of diverse groups at later time points, resulting in attrition bias (Green et al., 2022). Furthermore, exclusion biases can also result from inadequacies of neuroimaging equipment not designed with particular features in mind (e.g., poor data quality from electroencephalography (EEG) caps not designed for voluminous, curly, and/or tightly coiled hair types), contributing to a lack of diversity in FIT neuroimaging research samples (Green et al., 2022).

**Fig. 1:**
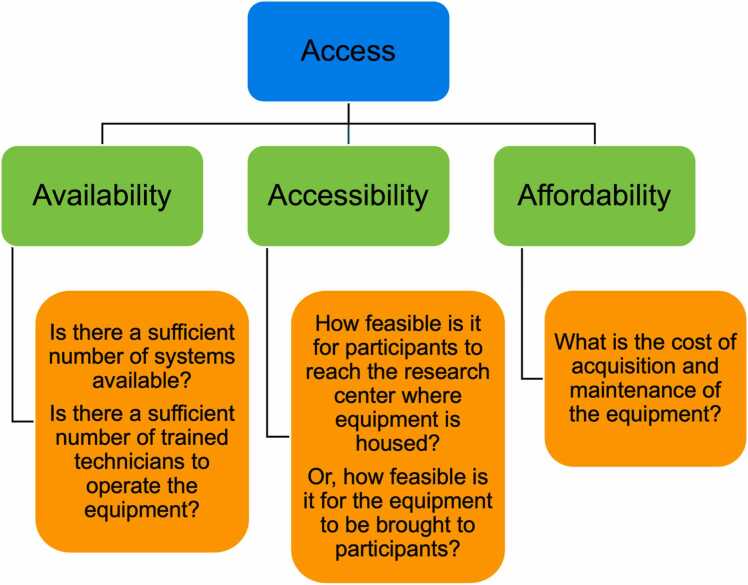
An illustrated Access Framework for Neuroimaging Research, modified from Penchansky and Thomas’s Access Framework for Health Care (1984). Availability, accessibility, and affordability are the three dimensions of access to neuroimaging technology discussed in this paper.

Environmental-level barriers to representation include systemic barriers, such as the limited availability of neuroimaging technologies in countries within the global majority. Even when resources are available, neuroimaging research has traditionally been geographically constrained to well-resourced sites. Compounded with the limited portability and adaptability of current neuroimaging technologies, it hinders researchers’ abilities to effectively reach marginalized populations and rural communities (Flores et al., 2017, Nicholson et al., 2015, Raphael et al., 2017). The lack of diversity among scientists at all levels of the research team may also contribute to limited recruitment, higher participant attrition rates (Green et al., 2022, Tzovara et al., 2021), and the distrust in science that marginalized communities may experience (Flores et al., 2017, Wallace and Bartlett, 2013). Lack of access to neuroimaging modalities is another factor limiting the representativeness of FIT research samples, especially those from lower-resourced or rural areas far from larger university or hospital institutions. Access for neuroimaging research (see Fig. 1) includes availability (i.e., availability/prevalence of neuroimaging systems and trained technicians), accessibility (i.e., feasibility for participants to travel to neuroimaging systems or for equipment to be brought to participants), and affordability (i.e., cost of acquisition/maintenance of equipment; modified from Penchansky and Thomas (1981). Addressing these systemic barriers is essential to making FIT neuroimaging research more inclusive and representative of the diverse populations it seeks to learn about and support with scientific knowledge.

This article facilitates a self-reflective discussion on inclusivity in FIT neuroimaging by examining historical and current obstacles and recent advancements toward achieving equitable representation of research participants across key neuroimaging modalities, including magnetic resonance imaging (MRI), magnetoencephalography (MEG), electroencephalography (EEG), functional near-infrared spectroscopy (fNIRS), and ultrasonography. It comprises the cumulative knowledge of researchers actively conducting research with diverse populations. Lastly, we acknowledge that this is an ongoing and complex discussion that requires continuous collaboration, innovation, and intentionality to achieve equitable representation of diverse populations in FIT neuroimaging research.

While we encourage reading this article in its entirety, the body of the article – *Modality-Level Obstacles and Ongoing Initiatives* – addresses similar obstacles across modalities. The article is designed so readers with an interest in a singular modality can read the introduction, followed by the corresponding modality-section to receive a comprehensive overview of challenges and efforts, and last, concluding thoughts in which we identify overarching directions for the field that transcend modality.^2^

## 2 Modality-level obstacles and ongoing initiatives

### 2.1 Magnetic resonance imaging

Magnetic resonance imaging (MRI) is an invaluable tool for researching in-vivo brain development throughout the FIT age continuum. It has high spatial but low temporal resolution, excellent soft tissue contrast, and can be used during natural sleep and wakefulness. However, it is contraindicated for individuals with non-removable ferromagnetic metal or other unsafe materials in or attached to body parts (Ghadimi and Sapra, 2024). It has been extensively utilized to research brain activation (functional MRI), volume and surface area (structural MRI), and integrity and organization of white matter tracts (diffusion MRI), among other useful brain measures (Bethlehem et al., 2022, Giedd et al., 1999).

#### 2.1.1 Availability, accessibility, and affordability

*Challenges.* A primary obstacle in FIT MRI research is the high cost of acquisition ($1 million US Dollars/Tesla) and maintenance, as well as lack of portability of the equipment (3 T scanner is ∼ 5–8000 kg; Foo et al., 2018). The cost of MRI machines results in near-zero MRI density in regions of the world that are medically underserved (Geethanath and Vaughan, 2019, Ogbole et al., 2018, World Health Organization, 2024), which is further supported by empirical evidence that the amount of MRI research in a country is related to local economic conditions (Duncan and Rae, 2024). Additionally, MRI machines in resource-limited contexts are usually located in large universities or medical centers and are often reserved for medical purposes, limiting the number of MRI facilities available for research. When these facilities are available to use for research, families from resource-limited or rural areas may require extensive resources (e.g., childcare, transportation) to facilitate travel to these centers (Jalloul et al., 2023, Zgierska et al., 2024). Further, many older infant and toddler MRI scans occurring during natural sleep are scheduled at night (Korom et al., 2022), which can pose an additional burden on families and limit who is able to participate in this research (Zgierska et al., 2024).

*Ongoing Initiatives.* No easy solution exists to mitigate the high cost and resources necessary to host and maintain high-resolution MRI scanners. Low-field (∼0.25–1.0 T; Marques et al., 2019) and ultra low-field MRI (∼<.1 T; Liu et al., 2021) have emerged as potential alternatives, reducing costs and infrastructure needs while improving portability and accessibility in lower-resource regions. For example, the FDA-approved portable Hyperfine Swoop imaging system (64mT) has shown promise for bedside clinical use and in research settings with FIT populations (Cawley et al., 2023, Deoni et al., 2021, Fite, 2021, Padormo et al., 2023) with emerging evidence of its feasibility in the Global South via the UNITY Project (Abate et al., 2024). Low-field MRIs are safer for individuals with metal implants, particularly when the B0 field is below 0.5 T, and cause less image degradation in this case than high-field MRI scanners (Kraus et al., 2014). Moreover, ultra-low field MRIs are portable and often feature "open-type" configurations, accommodating caregiver presence more easily during data collection. However, compared to high-field MRI, low-field MRIs differ in aspects fundamentally tied to field strength, such as T1 longitudinal relaxation values, chemical shifts, and acquisition time (Abate et al., 2024, Hori et al., 2021). The reduction in field strength also leads to a significant signal loss and a decrease in signal-to-noise ratio. Efforts are ongoing to train machine learning algorithms to mitigate resulting decreases in image resolution (Baljer et al., 2024, Lau et al., 2023). Yet, at the present moment, low-field machines only support anatomical and to some extent diffusion MRI (Liu et al., 2021), so other solutions are needed to improve the accessibility of MRI research. Even when high-field MRI scanners are available, steps can be taken to make them accessible to all participants. This includes coordinating and/or reimbursing expenses for travel, accommodation (especially for nighttime scans), and childcare (Zgierska et al., 2024). Budgeting for these family-friendly costs is important for investigators and funding agencies to consider as it may increase the financial resources necessary for the study.

#### 2.1.2 Misconceptions about safety and invasiveness

*Challenges*. Misconceptions about the safety and invasiveness of MRI procedures pose significant challenges (King et al., 2023). For example, families may not be aware that an MRI scan does not expose the participant to ionizing radiation. Caregivers may also assume that agreeing to participate in research involving a scan will require sedation and/or restraint of young children and/or the use of contrast agents, practices which are often used for medical purposes but not in a research setting (Balachandar et al., 2024, Copeland et al., 2021, Dean et al., 2014). Communities historically exploited in medical research may harbor fear and distrust of medicine and scientific research (Bazargan et al., 2021, Kennedy et al., 2007). This distrust may make recruiting participants from these communities difficult, as they may already be wary of the research team and methods. As noted earlier, some research suggests that individuals from marginalized groups are equally likely to participate in research when they are equitably recruited (Bradford et al., 2024, Fisher and Kalbaugh, 2011, Wendler et al., 2005). In the context of fetal MRI, additional barriers exist, as many individuals, even in the scientific community, mistakenly believe MRI scanning (even in the absence of sedation or gadolinium-based contrast agents) is unsafe for pregnant women (Maralani et al., 2023).

*Ongoing Initiatives.* It is the responsibility of the research team to address any misconceptions clearly during recruitment and study visits by effectively communicating that MRI does not involve harmful ionizing radiation, that no contrast agents or sedation will be used during the study, and that procedures are designed to ensure safety and comfort with ethical review board approval. Engaging with families early and providing thorough explanations in clear and understandable terms can build trust. Family-facing materials developed by researchers in collaboration with the community to explain the MRI process in an accessible way (see Table 1 for resources) play a significant role in dismantling misconceptions, reassuring participants and fostering a more inclusive research environment. Research teams should represent diverse backgrounds to promote feelings of familiarity and similarity among participants, which in turn may increase trust in science and research (Flores et al., 2017, Green et al., 2022, Wallace and Bartlett, 2013).

**Table 1:** Recommended resources for families on neuroimaging modalities.

**Modality**	**Title**	**Type**	**Source**	**Link**
MRI	What to Expect During your Study Visit?	Informational video	Maternal Influence on Neurodevelopment Lab, Virginia Tech, USA	Link
	fMRI Scanning at MIT: For Kids	Informational video	MIT Center for Minds, Brains and Machines (CBMM)	Link
	Neuroimaging for Toddlers	Informational video	Cape Universities Body Imaging Centre, University of Cape Town	Link
	Pluto and the MRI Rocket Ship Adventure	Storybook	Developmental Neuroimaging Lab, University of Calgary, Canada	Link
	Magnetic Resonance Imaging (MRI): Frequently Asked Questions	Informational Website	HEALthy Brain and Child Development Study	Link
EEG	Infant Electroencephalography (EEG) Visits	Informational video	Baby’s First Years Study (Request from Dr. Troller-Renfree)	Upon Inquiry for Research Purposes
	Electroencephalography: Frequently Asked Questions	Informational Website	HEALthy Brain and Child Development Study	Link
	Using Electroencephalography (EEG) to Learn About the Brain	Informational video	Plasticity in Neurodevelopment Lab, Northeastern University, USA	Link
fNIRS	Finn the Fox: Discovering the Brain with Red Light	Storybook	MIND in Development Lab, Smith College, USA	Link
	Have you ever heard of Functional Near-Infrared Spectroscopy?	Informational video	Frontiers for Young Minds	Link

#### 2.1.3 Sensory difficulties

*Challenges.* Clinically vulnerable populations may be more sensitive to the unfamiliar nature of the scanning environment. For example, individuals born prematurely or infants and toddlers on the autism spectrum may be more sensitive to noise (Germani et al., 2014, Guiraud et al., 2011, Kuhn et al., 2012, McMahon et al., 2012), touch (André et al., 2020, Mammen et al., 2015), and/or changes in their bedtime routines (for nighttime scans; Souders et al., 2017), which may limit successful MRI participation. The machine noises may be distressing to both young children (Philbin et al., 1996), who may be less likely to complete the scanning session, and their caregivers (King et al., 2023), who may be reluctant to expose their children to additional discomfort.

*Ongoing Initiatives.* A comprehensive approach to mitigating sensory risks before and during data collection is necessary for all FIT participants, but it is particularly essential for clinically vulnerable populations. Detailed inquiries into each child's sensory sensitivities and typical sleeping environment are crucial to tailoring the environment to maximize comfort. To acclimate the infant to potential sensory triggers before the visit, researchers can provide families with audio recordings of scanner noises (and a speaker if needed) and the same form of hearing protections that infants will wear during the scan. If multiple options are available, families can determine which type their child tolerates best. During the scan, acoustic noise is reduced as much as possible through the use of noise reduction pads, external headphones with active noise cancellation, playing white noise (Korom et al., 2022), and/or silent sequences (Aida et al., 2016, Barkovich et al., 2018). Placing MRI-safe weights around the head coil or on the scanner table can also reduce noise-related vibrations. Most infant scanning protocols include a trained assistant and/or the caregiver beside the scanner within arm's reach of the child during the scan to reassure the child, monitor their comfort and stop the session if required (Korom et al., 2022, Spann et al., 2023). Involving caregivers in the scanning procedure whenever possible provides caregivers with more agency and may make procedures more comfortable for children, especially those with sensory sensitivities. Families can also bring the child's favorite MRI-safe blanket or toys for additional comfort and familiarity. These measures enhance the feasibility of including vulnerable populations in neuroimaging studies by reducing sensory-related distress.

#### 2.1.4 MRI contraindications

*Challenges.* The strong magnetic field of MRI scanners requires strict safety regulations, often preventing participation if children or caregivers wear metal accessories (Cross et al., 2018). Hair accessories and jewelry which cannot be removed, like baby earrings, can prevent children from participating. Caregivers must also adhere to these protocols in order to be allowed to enter the scanning room with their child. Metal accessories (e.g., non-removable piercings, metal bead hair accessories, pins in head coverings), medical devices (e.g., cochlear implants, pacemakers, continuous glucose monitors, pins, and rods), and non-professional tattoos can prevent caregivers from entering the scanning room due to burn risk and risk of the strong magnetic field disrupting life-saving devices. These restrictions can disproportionately preclude participation from certain cultural groups, who may already be underrepresented in FIT neuroimaging research, especially when removal of accessories is impossible, uncomfortable, or violates cultural and religious norms.

*Ongoing Initiatives.* Early and clear communication about MRI contraindications is crucial. Informing caregivers starting 1–2 months before the scan that removing metal accessories is required for participation allows for necessary accommodations where possible. For example, families might schedule scans before ear piercing or avoid metal bead accessories. The research team can provide MRI-safe plastic earrings beforehand, so caregivers can replace them at home. At the visit, metal-free alternatives, like plastic pins for head coverings, should be made available. While some contraindications, like non-professional tattoos, cannot be addressed, informing caregivers in advance avoids surprises and allows for preparation, such as having another caregiver attend the session who may accompany the child into the scanner room.

### 2.2 Magnetoencephalography

Magnetoencephalography (MEG) directly measures the miniscule changes in magnetic field created by groups of firing neurons, allowing for non-invasive measurement of brain activity (Lopes da Silva, 2013). MEG provides high temporal resolution data comparable to EEG (sampling rates are typically right around 1200 Hz), while also offering high spatial resolution (Hedrich et al., 2017) and lifespan compliance, making it amenable to questions of functional brain development in FIT populations (Chen et al., 2019; Schwartz et al., 2010; Sheridan et al., 2010). MEG is relatively robust to motion, silent, and does not require sensors to make contact with the scalp, which enhances tolerance in young pediatric populations. The improved ease of use, ability to assess participants during tasks or sleep, and greater participant comfort make MEG an attractive technique for assessing brain function in diverse FIT populations, with few intractable targets for overcoming barriers to equitable science.

#### 2.2.1 Availability, accessibility, and affordability

*Challenges.* The most significant obstacle to utilizing MEG in diverse and representative populations is its availability and affordability, largely due to the cost of establishing a system. One of the most expensive aspects of MEG is the need for a shielded space to measure the tiny magnetic fields generated by brain activity, on the order of femtoTesla compared to the several order of magnitudes stronger magnetic field of the Earth, as well as those created by electric circuits. Magnetically shielded rooms can cost millions of dollars to install on top of the cost of the other hardware required. MEG systems fall into two general types: large stationary arrays of superconducting quantum interference devices (SQUIDs) (Hari and Salmelin, 2012), and more flexible, often wearable arrays of optically pumped magnetometers (OPM systems; Hill et al., 2020). SQUIDs require constant cooling using cryogens, much like MRI scanners, making them costly to install and maintain. Also, because signal strength is solely dependent on distance from the source of the signal, for applications to FIT populations specific pediatric systems are often needed, e.g., BabySQUID (Okada et al., 2006). In terms of accessibility, because of the high cost to establish MEG systems, these systems are generally established at well-resourced academic and medical institutions. Thus, it is challenging for individuals in under-resourced or rural areas to participate in this type of research without dedicating resources towards accessibility (Duncan and Rae, 2024). It is also important to highlight that in order to achieve high spatial resolution, that is, accurate source localization, it is ideal to also obtain a high resolution structural MRI, which additionally contributes to issues of accessibility for researchers, particularly those at smaller, non-academic health center associated institutions.

*Ongoing Initiatives.* OPM systems have the potential to improve upon the limited access of more traditional SQUID MEG systems because of their lower cost and increasing availability from commercial companies. OPM technology is evolving daily, as systems become smaller, more affordable, and more robust to movement. Additionally, although researchers are far from being able to apply MEG outside of a magnetically shielded room, work is being done to move towards measurement of magnetic fields around the head in ambient fields (Limes et al., 2020), which would open this method up to more populations, perhaps even allowing researchers to go to participant communities and homes to conduct their research. Despite all of the characteristics of MEG that make it inherently amenable to research in developing populations from all backgrounds, there have been very few studies that have leveraged these positive attributes to ensure representation in our datasets.

#### 2.2.2 Misconceptions about safety and invasiveness

*Challenges.* Similarly to other neuroimaging modalities, there exist public misconceptions about the safety and invasiveness of MEG procedures. As a less widespread modality, it may also be confused for similarly named techniques such as MRI or EEG (see MRI and EEG 2.1.2, 2.3.4 on obstacles related to *Misconceptions about safety and invasiveness*).

*Ongoing Initiatives.* Efforts to mitigate misconceptions related to the safety and invasiveness of procedures are similar to those in other modalities, most notably the use of family-friendly educational materials (see MRI and EEG 2.1.2 and 2.3.4 sections on ongoing efforts related to *Misconceptions about safety and invasiveness*). See Table 1 for examples of family-friendly educational materials.

### 2.3 Electroencephalography

Electroencephalography (EEG) is a noninvasive, passive neuroimaging method that uses small sensors, called electrodes, to record the electrical activity from the brain. Within FIT populations, EEG is primarily used postnatally for infants and toddlers, although new advances are making it possible to measure brain activity *in utero* (Thaler et al., 2000). EEG offers excellent temporal resolution, with precise millisecond measurements, but has lower spatial resolution compared to many other neuroimaging modalities (Barry-Anwar et al., 2020). EEG systems are relatively cost-effective and portable with fewer contraindications compared to other neuroimaging technologies (Barry-Anwar et al., 2020).

#### 2.3.1 Availability, accessibility, and affordability

*Challenges.* EEG is one of the more affordable and accessible neuroimaging methods as compared to some of the other neuroimaging technologies discussed in this article. EEG system affordability varies widely, largely dependent on the density of electrodes, with lower-density systems up to 32 electrodes costing hundreds to a few thousands of US dollars and high-quality lab or hospital systems costing $100,000 or more. Traditionally, EEG research has required participant travel to the research center, which requires allocation of additional resources. Recently, many researchers have focused on improving EEG accessibility through increased portability of EEG systems, allowing data collection to happen in various remote locations such as participant’s homes and schools (Bhavnani et al., 2022, Troller-Renfree et al., 2021). Troller-Renfree and colleagues (2021) have summarized parameters to consider when selecting a portable EEG system. The signal to noise ratio of mobile systems is worse than lab-based systems, necessitating longer recording times. In addition, due to constraints on energy required and amplifier size, this work has primarily focused on lower-density systems with a maximum of 32 electrodes. Additional work is necessary to increase the portability and therefore accessibility of high-density EEG systems. While empirical evidence suggests that EEG research is more prevalent in countries with lower economic resources compared to other modalities such as MRI, researchers can always be looking to improve the availability of EEG in lower-resource research contexts (Andrews et al., 2022; Duncan and Rae, 2024).

*Ongoing Initiatives.* Researchers requiring more affordable EEG systems may opt for lower-density systems which tend to be more cost-effective. Developers who are building and refining these systems could in parallel work on decreasing the cost of high-density systems. With regards to accessibility, one potential solution is increased resource allocation to reduce barriers to participant travel to research centers providing/reimbursing transportation and childcare for visits (see section 3.5 on *High resource requirements for inclusive research*). Another solution to accessibility is improving the portability of EEG systems, especially higher-density systems for which more progress is required. Suggestions for improving the availability of EEG systems and trained technicians are discussed in section 3.8 on *Increasing the global density of neuroimaging technologies.*

#### 2.3.2 Accommodating diverse hair texture phenotypes

*Challenges.* The use of EEG as a neuroimaging modality for diverse populations has been severely limited by two major challenges related to the non-inclusive cap/net designs (Mlandu et al., 2024, Adams et al., 2024). First, most widely-available caps/nets are not designed for denser, thick, or voluminous hair as they provide limited room for hair underneath the cap/net. Second, most electrodes are rather short or low profile. This prohibits them from reaching the scalp when hair is dense, thick or voluminous. These design issues commonly lead to unintended systemic racial biases in EEG research as individuals who identify as Black and/or Latine/x are more likely to have these hair phenotypes (Andrews and Swaine, 2022, Garcini et al., 2022, Girolamo et al., 2023, Green et al., 2022, Mlandu et al., 2024, Norton et al., 2021; Penner et al., 2023; Webb et al., 2022). Additionally, recent research suggests that for gel-based EEG systems, increased hair volume may affect signal amplitude of Event-Related Potentials, which can be mitigated by controlling for gel volume (Lees et al., 2024). However, this study was performed on adults, so its application to infant and toddler populations remains untested and further research is needed.

*Ongoing Initiatives. High Density Systems.*Mlandu and colleagues (2024) validated modified high-density 128-channel saline-based EEG nets from Magstim Electrical Geodesics, Inc. (Whitland, UK) in 6-to-11-month-old infants with curly and coily hair types (Adams et al., 2024, Mlandu et al., 2024). These modified nets, now commercially available, replace shorter pedestals encasing electrodes with pedestals that are 2.8 mm taller and made of a more rigid plastic material, resulting in higher channel retention and lowered impedances (Mlandu et al., 2024). Electrodes in both nets are encased in a non-closed system of stretchy elastomers, providing increased room for more voluminous hair. *Low-Density Systems (<32 electrodes)*. Low-density systems may facilitate more scalable EEG data collection. Etienne and colleagues (2020) have designed innovative *Sevo electrodes* for low-density EEG, consisting of an electrode on a clip, that are individually braided into curly or coily hair in a 10–20 EEG arrangement, “harnessing” the properties of curly hair. While these electrodes have been piloted in pediatric epilepsy patients (Kwasa et al., 2021), additional considerations (e.g., clip size, amount of hair needed to secure electrodes, increased time required for manual electrode placement) are necessary to adapt these electrodes to infant and toddler populations. Other low-density EEG systems with electrodes embedded in a closed cap do not require manual placement but provide more limited room to stretch over thicker curly or coily hair. Additionally, most lower-density systems use gel as a conducting agent, which may be less preferable for some participants as it may require hair washing after data collection. Emotiv (San Francisco, USA) produces 32-electrode caps that use a saline conducting agent, which may be preferable for families where hair washing may be time-consuming and stressful. Emotiv caps can also be configured with taller electrodes, similarly to the high-density solution mentioned earlier. While promising, further validation of this system in infant populations with curly or coily hair is needed, especially as the current closed-system cap design leaves limited room for more voluminous hair textures, and infant-sized caps are custom ordered as of writing. Other viable solutions include dry electrodes which do not require the use of a conducting agent, which are often taller and more stiff, which helps with application to more dense, voluminous hair (Troller-Renfree et al., 2021).

#### 2.3.3 Researcher understanding of cultural hair practices

*Challenges.* Gaps in researcher understanding surrounding cultural preferences and routines for hair texture, washing, and styling is another source of bias limiting inclusivity of diverse populations in FIT neuroimaging research (Adams et al., 2024). As researchers, it is important to acknowledge the cultural significance of hair for many families from a variety of backgrounds. It is also important that researchers recognize the historic and ongoing discrimination surrounding hair texture or style that may affect marginalized communities (Essien and Wood, 2021, Henning et al., 2022, Mbilishaka and Apugo, 2020). Families may traditionally choose to style their children’s hair in longer-lasting hairstyles for practical reasons as well as a means of self- or cultural-expression. When scheduling neuroimaging sessions, many researchers request that children wear their hair “down,” which may be impossible for some styles (e.g., locs), burdensome (e.g., removing braids), or make families uncomfortable due to existing stigmas around unstyled hair appearing “frizzy” or “messy” (Adams et al., 2024). Additionally, many intricate, protective, and texture-altering hairstyles for young children require substantial time and effort with a high monetary cost. These hairstyles are often intended to last weeks or months. They are also often styled around children’s birthdays and/or holidays/big events (Adams et al., 2024). This is particularly relevant in FIT neuroimaging research in which visits are often scheduled around birthday milestones. Further, some cultural or religious groups may traditionally shave, cut, or cover their infant/toddler’s hair at certain age milestones. Communication with families about hair at milestone ages is important as it may impact the data collection process. All in all, requesting that families come to the research visit with their child’s hair “down” or unencumbered may unintentionally place a burden on families that wish to participate (wasting time, effort, and money) or may result in families declining participation (Adams et al., 2024). In addition to styling requests, it is common for researchers to request that participants’ hair is “clean” before the data collection session and recommend that hair is washed again after the visit to remove any remaining residue or media. These requests may also be placing an unintentional burden on participants and ultimately bias participation. First, the term “clean” can sometimes be a loaded term for families as it may unintentionally perpetuate stereotypes that less-frequently washed or styled hair, which is more common for curly/coily hair textures is “unclean” (Essien and Wood, 2021, Henning et al., 2022, Mbilishaka and Apugo, 2020). Additionally, hair washing and detangling, whether before or after data collection, can be an incredibly time-intensive process, which places a burden on the family. Furthermore, asking families to wash their child’s hair multiple times in close proximity may be burdensome, uncomfortable, or result in hair or scalp dryness for participants with denser, curly, and/or coily hair textures (Adams et al., 2024). Finally, families living in informal settlements or rural areas may have limited access to running water, making the repeat washing of hair a burden.

*Ongoing Initiatives.* Improved researcher education and respect about cultural significance, preferences, and routines surrounding hair is a key step in closing this gap in understanding. Importantly, while the precedent may be to request that hair is “clean” before EEG recordings, basic methodological work is required to investigate how/if increased scalp oils due to not pre-washing hair impacts the EEG signal (Adams et al., 2024). In fact, some researchers have reported that applying a commonly used hair conditioner with insulating ingredients actually prevents hair tangling and makes net placement and removal more comfortable (Mlandu et al., 2024), although the impact on the EEG signal remains uninvestigated. Culturally responsive communication before, during, and after EEG collection is essential in improving inclusivity of diverse populations in EEG research and examples are now available in English and Spanish and highlighted in Table 2 (Adams et al., 2024).

**Table 2:** Recommended resources for scientists.

a. History of Exclusionary Practices in Psychological and Neuroimaging Research			
**Title**	**Authors**	**Year**	**Link**
An Antiracist Research Framework: Principles, Challenges, and Recommendations for Dismantling Racism Through Research	Goings, T. C., Belgrave, F. Z., Mosavel, M., & Evans, C. B. R.	2023	DOI
Confronting Racially Exclusionary Practices in the Acquisition and Analyses of Neuroimaging Data	Ricard, J. A., Parker, T. C., Dhamala, E., Kwasa, J., Allsop, A., & Holmes, A. J.	2023	DOI
Implementing Neuroimaging and Eye Tracking Methods to Assess Neurocognitive Development of Young Infants in Low-and Middle-Income Countries	Katus, L., Hayes, N. J., Mason, L., Blasi, A., McCann, S., Darboe, M. K., de Haan, M., Moore, S. E., Lloyd-Fox, S., & Elwell, C. E.	2019	DOI
Psychology's Contributions to Anti-Blackness in the United States within Psychological Research, Criminal Justice, and Mental Health	Auguste, E., Bowdring, M., Kasparek, S. W., McPhee, J., Tabachnick, A. R., Tung, I., Scholars for Elevating Equity and Diversity (SEED), & Galán, C. A.	2023	DOI
Psychological Science Is Not Race Neutral	Dupree, C. H., & Kraus, M. W.	2022	DOI
Racial Disparities in Adversity During Childhood and the False Appearance of Race-Related Differences in Brain Structure	Dumornay, N. M., Lebois, L. A. M., Ressler, K. J., & Harnett, N. G.	2023	DOI
Racial Disparities in EEG Research and Their Implications for our Understanding of the Maternal Brain	Penner, F., Wall, K. M., Guan, K. W., Huang, H. J., Richardson, L., Dunbar, A. S., Groh, A. M., & Rutherford, H. J. V.	2023	DOI
Racial Inequality in Psychological Research: Trends of the Past and Recommendations for the Future	Roberts, S. O., Bareket-Shavit, C., Dollins, F. A., Goldie, P. D., & Mortenson, E.	2020	DOI
Structural Racism in Neuroimaging: Perspectives and Solutions	Parker, T. C., & Ricard, J. A.	2022	DOI
The Inequitable Burden of Open Science	Fox Tree, J., Lleras, A., Thomas, A., & Watson, D.	2022	Link

b. Roadmaps and Recommendations for More Inclusive Science			
**Title**	**Authors**	**Year**	**Link**
A Causal Framework for Cross-Cultural Generalizability	Deffner, D., Rohrer, J. M., & McElreath, R.	2022	DOI
A Unified Approach to Demographic Data Collection for Research With Young Children Across Diverse Cultures	Singh, L., Barokova, M. D., Baumgartner, H. A., Lopera-Perez, D. C., Omane, P. O., Sheskin, M., Yuen, F. L., Wu, Y., Alcock, K. J., Altmann, E. C., Bazhydai, M., Carstensen, A., Chan, K. C. J., Chuan-Peng, H., Dal Ben, R., Franchin, L., Kosie, J. E., Lew-Williams, C., Okocha, A., Reinelt, T., Schuwerk, T., Soderstrom, M., Tsui, A. S. M., & Frank, M. C.	2023	DOI
Addressing Racial and Phenotypic Bias in Human Neuroscience Methods	Webb, E.K., Etter, J.A., & Kwasa, J.A.	2022	DOI
Advancing Health Equity: A Guide to Language, Narrative, and Concepts	American Medical Association Center for Health Equity	2021	Link
Assessing Field Standard Practices for Incorporating Black individuals in EEG Research	Brown, L.	2023	Link
Authentic Engagement of Patients and Communities can Transform Research, Practice, and Policy	Woolf, S. H., Zimmerman, E., Haley, A., & Krist, A. H.	2016	DOI
Cortivision fNIRS Tutorial - Hair Management for Better Signal Quality	Cortivision	2023	Link
Current Reporting Practices in Human Neuroscience Research	Gard, A. M. S., Shariq, D., Albrecht, A., Lurie, A., Kim, H. C., Mitchell, C., & Hyde, L. W.	2024	DOI
Demographic Reporting and Reporting Phenotypic Exclusions in fNIRS	Kwasa, J., Peterson, H. M., Karrobi, K., Jones, L., Parker, T., Nickerson, N., & Wood, S.	2023	DOI
Equity, Diversity, and Inclusion in Developmental Neuroscience: Practical Lessons from Community-Based Participatory Research	La Scala, S., Mullins, J. L., Firat, R. B., Emotional Learning Research Community Advisory Board, & Michalska, K. J.	2023	DOI
Feasibility of Assessing Brain Activity Using Mobile, In-Home Collection of Electroencephalography: Methods and Analysis	Troller-Renfree, S. V., Morales, S., Leach, S. C., Bowers, M. E., Debnath, R., Fifer, W. P., Fox, N. A., & Noble, K. G.	2021	DOI
Flux Diversity Session 2023: Lessons Learned from ABCD JEDI	Uddin, L., Bodison, S. C., & Cardenas-Iniguez, C.	2023	DOI
fNIRS for Tracking Brain Development in the Context of Global Health Projects	Blasi, A., Lloyd-Fox, S., Katus, L., & Elwell, C. E.	2019	DOI
Fostering Inclusion in EEG Measures of Pediatric Brain Activity	Adams, E. J., Scott, M. E., Amarante, M., Ramírez, C. A., Rowley, S. J., Noble, K. G., & Troller-Renfree, S. V.	2024	DOI
Geographical and Economic Influences on Neuroimaging Modality Choice	Duncan, N. W. & Rae, C. L.	2024	DOI
Hair, Community, & EEG Webinar	Richardson, L. & Black in Neuro	2021	Link
Hair Me Out: Highlighting Systematic Exclusion in Psychophysiological Methods and Recommendations to Increase Inclusion	Louis, C. C., Webster, C. T., Gloe, L. M., & Moser, J. S.	2022	DOI
Inclusive Language Guide, Second Edition	American Psychological Association	2023	Link
Increasing Diversity in Developmental Cognitive Neuroscience: A Roadmap for Increasing Representation in Pediatric Neuroimaging Research	Garcini, L. M., Arredondo, M. M., Berry, O., Church, J. A., Fryberg, S., Thomason, M. E., & McLaughlin, K. A.	2022	DOI
Magstim EGI Webinar: Inclusive HD-EEG Research for All People	Hudac, C. & Magstim EGI	2023	Link
Recommendations for the responsible use and communication of race and ethnicity in neuroimaging research	Cardenas-Iniguez, C. & Robledo Gonzalez, M.	2024	DOI
Systemic Racism in EEG Research: Considerations and Potential Solutions	Choy, T., Baker, E., & Stavropoulos, K.	2022	DOI
The Effect of Hair Type and Texture on Electroencephalography and Event-Related Potential Data Quality	Lees, T., Ram, N., Swingler, M. M., & Gatzke-Kopp, L. M.	2024	DOI
The Promise and Pitfalls of a Strength-Based Approach to Child Poverty and Neurocognitive Development: Implications for Policy	DeJoseph, M. L., Ellwood-Lowe, M. E., Miller-Cotto, D., Silverman, D., Shannon, K. A., Reyes, G., Rakesh, D., & Frankenhuis, W. E.	2024	DOI
Untangling bias: Racial and Phenotypic Bias in Neuroimaging Methods Must Be Addressed	Kwasa, J.	2024	DOI
Whose Signals are Being Amplified? Toward a more Equitable Clinical Psychophysiology	Bradford, D. E., DeFalco, A., Perkins, E. R., Carbajal, I., Kwasa, J., Goodman, F. R., Jackson, F., Richardson, L.N.S, Woodley, N., Neuberger, L., Sandoval, J.A., Huang, H.L., & Joyner, K. J.	2024	DOI
Working Toward Anti-Racist Perspectives in Attachment Theory, Research, and Practice	Stern, J. A., Barbarin, O., & Cassidy, J.	2022	DOI

#### 2.3.4 Misconceptions about safety and invasiveness

*Challenges.* Like most neuroimaging techniques, there exists misconceptions about the safety and invasiveness of EEG procedures (Shivji et al., 2011, Tekin et al., 2020; see MRI section 2.1.2 on obstacles related to *Misconceptions about safety and invasiveness*). Individuals may be unaware that EEG does not require sedation or restraint and that it records the very small electrical signals already happening in the brain rather than applying or exposing participants to electrical stimulation.

*Ongoing Initiatives.* This challenge can be resolved by providing clear educational materials and engaging in effective communication that demystifies EEG procedures, emphasizing that EEG is non-invasive, does not involve sedation or restraint, and only records natural brain activity without applying electrical stimulation. When applicable, researchers can differentiate EEG research procedures from clinical EEG assessments that may require abrasion of the scalp or other more involved procedures not used in the research environment. Other ways to mitigate misconceptions are covered in the corresponding MRI section, including early and clear communication, use of family-friendly educational materials, and increased diversity of research teams. See Table 1 for examples of family-friendly educational materials.

#### 2.3.5 Sensory difficulties

*Challenges.* Infants and toddlers with neurodevelopmental disorders, such as autism spectrum disorders and attention-deficit hyperactivity disorders, who may exhibit lower focus levels, higher arousal, restlessness, and hypersensitivity, or with other sensory processing sensitivities, may find the experimental procedures to be aversive and struggle with the experimental procedures (Kaku et al., 2024).

*Ongoing Initiatives.* EEG researchers need to continuously read participant and caregiver cues and match the pace that is best for the participant, especially those with sensory sensitivities. However, there is no “one size fits all” procedure. Allowing caregivers to help wherever possible provides caregivers with more agency and may make procedures more comfortable for participants. A second researcher in charge of making participant families comfortable throughout the procedures who has distracting toys (e.g., bubbles, rain sticks) or engaging video clips on hand may be helpful (Hoehl and Wahl, 2012). For those using wet electrodes with saline solution, warming the solution to a safe temperature may make the capping process more enjoyable. For older infants/toddlers, applying an old unusable EEG cap to caregivers or a stuffed animal first and/or allowing children to tactilely explore an unusable cap can make the procedures feel less novel and scary. For older infants and toddlers who may pull at the EEG cap, it may be helpful to have infant/toddler-sized socks that participants can wear on their hands as mittens to mitigate any damage to equipment or scratching of the participant’s head. An alternate strategy is to provide the child with a smaller toy, such as a teething toy, to occupy their hands throughout the procedure.

### 2.4 Functional near-infrared spectroscopy

Functional near-infrared spectroscopy (fNIRS) uses near-infrared light to quantify changes in concentrations of oxyhemoglobin (HbO_2_) and deoxyhemoglobin (HHb) in cortical brain regions (Hoshi, 2016). fNIRS is silent, portable, quick to set up, highly tolerant to movement artifacts, not subject to electrical interference, and with recent technological advancements, becoming wearable and lightweight (Bulgarelli et al., 2023). As such, fNIRS has been increasingly chosen by developmental researchers as a non-invasive method for studying the developing brain (Gervain et al., 2023, Lloyd-Fox et al., 2010, Wilcox and Biondi, 2015). Moreover, diffuse optical tomography (DOT), an advanced version of the standard fNIRS system, has recently been applied in developmental populations (Frijia et al., 2021), providing whole head coverage (Collins-Jones et al., 2024), and offering spatial resolution comparable to MRI (Eggebrecht et al., 2012).

#### 2.4.1 Availability, accessibility, and affordability

*Challenges.* Although fNIRS is comparatively more affordable than other neuroimaging techniques, with widely used systems priced between $40,000 and $130,000 US dollars, it still requires advancements in availability, affordability, and accessibility, particularly in under-resourced communities. Its relatively recent introduction also means that there has been less time for the implementation of technological improvements that enhance the feasibility for diverse populations and to popularize the methodology globally, compared to other modalities, like EEG, MRI, or ultrasound.

*Ongoing Initiatives.* Significant efforts have been made to facilitate the transition of fNIRS technology from lab-based settings to real-world or naturalistic environments, including clinical settings (Uchitel et al., 2023), educational settings (Zhan et al., 2024), and homes (e.g., the PIPKIN project at the University of Cambridge. Additionally, progress is being made in collecting fNIRS data in global majority countries (Blasi et al., 2019, Jasińska and Guei, 2018), including the “BRIGHT” project in the Gambia (Katus et al., 2019, The BRIGHT project team, 2019), the “BEAN” project in Bangladesh (Perdue et al., 2019) and the “INDIA” project in India (Wijeakumar et al., 2019). Given that fNIRS technology does not require specially built infrastructure to collect high-quality data and is portable, wearable, and robust to motion artifacts, it has the potential to become a preferred option for studying early cortical brain development in lower-resource regions and among global majority populations.

#### 2.4.2 Accommodating diverse hair texture phenotypes

*Challenges.* Close coupling between the optode and the scalp is essential for obtaining high-quality fNIRS measurements (Scholkmann et al., 2014). However, hair textures, styles, and follicle density factors may present obstacles for fNIRS technology when working with participants who have thick hair and/or high follicle densities (Khan et al., 2012, Orihuela-Espina et al., 2010). As a consequence, hair density can obstruct the signal and interfere with direct optode placement on the scalp (Kwasa et al., 2023; Kwasa, 2024; Scholkmann et al., 2014; Strangman et al., 2002), resulting in reduced data quality (Khan et al., 2012) and up to 20–50 % signal loss (Koizumi et al., 1999), alongside phenotypic biases (Khan et al., 2012).

*Ongoing Initiatives.* Specialized data collection methodologies and adjustments to fNIRS hardware are necessary to better accommodate variability in hair texture and follicle density. A key initial step in addressing these biases is to collaborate with community members with these phenotypes (Parker and Ricard, 2022). Promising examples come from Romeo and colleagues who work with Black hair stylists and students to identify best practices for hair braiding in children participating in fNIRS data collection (Shih, 2023) or using combing techniques to move the hair away from underneath the optodes (Orihuela-Espina et al., 2010). To address the biases with fNIRS hardware, Kerr-German and colleagues worked with Black university students to improve the fit and functionality of the fNIRS cap for Black participants across development (Gratas, 2024, Honaker, 2023, December 6). Similarly, fNIRS engineers and researchers are making significant strides in developing specially designed optode attachments, such as those resembling a “brush” (Khan et al., 2012) or “mini comb” (Crawford et al., 2024) which can part hair to improve scalp-optode coupling and signal-to-noise ratios for all participants. Additionally, some researchers have developed a “protruding light guide” to minimize hair-reflected light (Vidal-Rosas et al., 2023; Yamada et al., 2015). Despite these efforts, most of this work has been conducted in adult populations, highlighting the need to further apply these advances to infant and toddler fNIRS research. Researchers can and are encouraged to push fNIRS manufacturers to prioritize the development of more inclusive technology while collaborating with participants from these communities to optimize fNIRS for inclusive practices.

#### 2.4.3 Accommodating diverse hair and skin pigmentation phenotypes

*Challenges.* Poor signal quality is a major contributor to data exclusion (Baek et al., 2023) from participants with darker skin and hair in research studies, thereby contributing to an exclusion bias in fNIRS samples (Orihuela-Espina et al., 2010). Specifically, the total attenuation coefficient of light in human hair increases with decreasing wavelength and increased pigmentation, with black hair exhibiting greater attenuation compared to blond, gray, and light brown hair (Kharin et al., 2009). As a result of increased light absorption in pigmented hair and skin, the precision of hemodynamic response measurements can be compromised, as many models estimating light transport through the scalp, skull, and biological tissue do not consider variations in skin pigmentation (Kwasa et al., 2023, McIntosh et al., 2010, Pringle et al., 1999). While fNIRS technology may be designed under the assumptions that tissue is optically homogeneous and the differential pathlength is invariable across skin tones, in actuality, some layers of the skin are optically heterogenous with melanin primarily absorbing NIR light in the epidermis and hemoglobin in the dermis (Kwasa et al., 2023). Higher melanin concentration in more pigmented hair and skin results in a systematic, nonlinear attenuation of the signal which likely underestimates relative changes in oxygenation (Kwasa et al., 2023).

*Ongoing Initiatives.* Despite the potential for fNIRS technology’s wide use, there has been limited ongoing research into how variations in skin and hair pigmentation may influence the accuracy and interpretability of fNIRS measurements in FIT populations. Most foundational work on pigmentation-related signal quality issues—such as the studies cited above—has been conducted in adult cohorts or animal models, leaving their applicability to FIT research uncertain. What we know is that newborns and young infants have thinner skulls, scalps, and less hair compared to adults, resulting in a threefold increase in light penetration into the cortex (Gervain et al., 2011). However, to our knowledge, no studies have systematically examined how gradual changes in skin and hair pigmentation affect signal power in FIT populations. Another significant limitation of most fNIRS studies is the lack of demographic information reporting, with virtually none providing details on skin pigmentation levels (e.g., using the Fitzpatrick scale, which ranges from 1 for very light to 6 for very dark skin) (Kwasa et al., 2023). Field-wide initiatives, such as reporting on racial and ethnic distribution, measuring and reporting on skin pigmentation, and analyzing the effects of pigmentation on fNIRS signal power, are necessary to draw just and accurate conclusions about FIT brain development. Ultimately, if empirical work confirms the hypothesis that signal power is attenuated by increased skin and/or hair pigmentation in FIT populations, researchers and developers must enhance fNIRS equipment and/or software to correctly account for varying pigmentation on the signal. They might look to the example of pulse oximetry, a similar technology whose limitations in deriving accurate optically derived readings across varying levels of skin pigmentation have recently been scrutinized, with stakeholders currently working on potential solutions (Al-Halawani et al., 2023, Kwasa et al., 2023).

#### 2.4.4 Researcher understanding of cultural hair practices

*Challenges.* fNIRS, similarly to EEG, requires participants to wear a fitted, specialized cap to record neural activity, and therefore, subject to biases created by gaps in research understanding surrounding cultural preferences and routines for hair texture, washing, and styling (see EEG section 2.3.3 on obstacles related to *Gaps in research understanding of cultural hair practices*). However, unlike EEG, fNIRS does not require the use of wet media and therefore does not require washing hair post-data collection.

*Ongoing Initiatives.* Similar to EEG, a keystone to mitigating sources of bias in fNIRS research is improved researcher education and respect of the cultural significance, preferences, and routines surrounding hair. Culturally responsive communication before, during, and after fNIRS data collection is another key step in improving the inclusivity of diverse populations in fNIRS research. Researchers can adapt the five-item framework from Adams and colleagues (2024) on approaching culturally responsive communication and visit scheduling for EEG for fNIRS studies. fNIRS researchers can also collaborate with hair stylists and students in the communities they serve to identify best practices for hair braiding for fNIRS collection (Shih, 2023). For instance, researchers conducting the “BRIGHT” project in the Gambia hired hair stylists to redo hair styles, such as braids, that were taken down for data acquisition (Lloyd-Fox et al., 2023). Additional resources on conducting culturally responsive fNIRS research are included in Table 2.

#### 2.4.5 Misconceptions about safety and invasiveness of procedures

*Challenges.* Like most neuroimaging modalities, mistrust and misconceptions surrounding safety and invasiveness of fNIRS procedures are highly prevalent, which may be greater in marginalized populations (see MRI and EEG 2.1.2, 2.3.4 on obstacles related to *Misconceptions about safety and invasiveness*).

*Ongoing Initiatives.* This obstacle can be resolved by providing clear educational materials and engaging in effective communication that demystifies fNIRS study procedures, while emphasizing that fNIRS is non-invasive and does not involve sedation or restraint (see MRI and EEG 2.1.2, 2.3.4 on ongoing efforts related to *Misconceptions about safety and invasiveness*).

#### 2.4.6 Sensory difficulties

*Challenges.* Similar to EEG, fNIRS requires that participants wear a fitted, specialized cap to record neural activity. Consequently, infant and toddler participants experiencing sensory processing difficulties may find the experimental procedures aversive (see EEG section 2.3.5 on obstacles related to *Sensory difficulties*).

*Ongoing Initiatives.* Since fNIRS and EEG share similar sensory experiences (with the exception of EEG potentially involving wet medium), many strategies developed over the years to mitigate sensory challenges in EEG can also be applied to fNIRS research (see EEG section 2.3.5 on ongoing efforts related to *Sensory difficulties*). In addition, some fNIRS companies are working towards creating bands instead of caps, which reduces the feeling of constriction on the participant’s head, although it sacrifices some coverage. Bulgarelli and colleagues are also working to personalize fNIRS data acquisition set-up for neurodiverse children by collecting parents’ feedback on how to better improve fNIRS testing sessions, aiming to mitigate childrens’ discomfort and yield high-quality data (Serino et al., 2023).

### 2.5 Ultrasonography

Fetal ultrasonography is an essential component of standard obstetric practice and is an invaluable tool in fetal neuroimaging research, providing data on fetal head biometry (Kocevska et al., 2018, Napolitano et al., 2016), midbrain growth (Mappa et al., 2024), and region-specific neurodevelopment patterns (Paules et al., 2021). Postnatal cranial ultrasonography is considered the standard of care for imaging preterm-born infants up to 12 months of age in global minority countries (Hand et al., 2020). It has also been utilized in developmental neuroscience research to gain insights into the precise timing of disrupted maturation (Cuzzilla et al., 2022, Cuzzilla et al., 2023, Fox et al., 2014, Hou et al., 2020); measure ventricular and brain volumes (Benavente-Fernandez et al., 2017; Benavente-Fernández et al., 2021); and quantify early brain growth as a marker of long-term development (Beunders et al., 2022, Hou et al., 2020, Ruiz-González et al., 2023). Ultrasonography is cost-effective, diagnostically useful, widely available, can be portable, and allows serial bedside imaging (Cuzzilla et al., 2018, Guillot et al., 2020, Hwang et al., 2022, Vo Van et al., 2022). Since ultrasonography is a widely used clinical neuroimaging technique in obstetrics and neonatology for assessing brain growth, injuries, and lesions, it encounters fewer obstacles in breaking down barriers to achieving equitable and inclusive science in fetal and infant neuroimaging research, such as misconceptions about its safety and invasiveness.

#### 2.5.1 Availability, accessibility, and affordability

*Challenges.* A primary obstacle to more widespread adoption of ultrasounds in FIT research is the limited availability of hospital-grade ultrasound equipment and trained sonographers in lower-resource contexts (Bagayoko et al., 2014, Holmlund et al., 2017, Nathan et al., 2017), where socioeconomic challenges in accessing clinical obstetric care are often prevalent. Women in regions in the Global South, like sub-Saharan Africa, may lack or have limited access to any ultrasound scans throughout their pregnancy (Aliyu et al., 2016, De Bernis et al., 2016, Luntsi et al., 2021, Wanyonyi et al., 2017).

*Ongoing Initiatives.* Portable ultrasound machines have been developed (Nelson and Narula, 2013) to address the shortage of prenatal ultrasounds in global majority countries (Becker et al., 2016). Portable ultrasounds, which cost as little as $2000 US dollars (Tang et al., 2022), can enable local research teams with more limited budgets to address research questions that are directly relevant to their communities they serve (e.g., effects of infection and malnutrition on fetuses). Further efforts are still needed to expand access in lower-resource settings, ultimately enhancing scientific understanding on fetal brain development and facilitating early detection of neurological complications.

## 3 Overarching directions for FIT neuroimaging

**Fig. 2:**
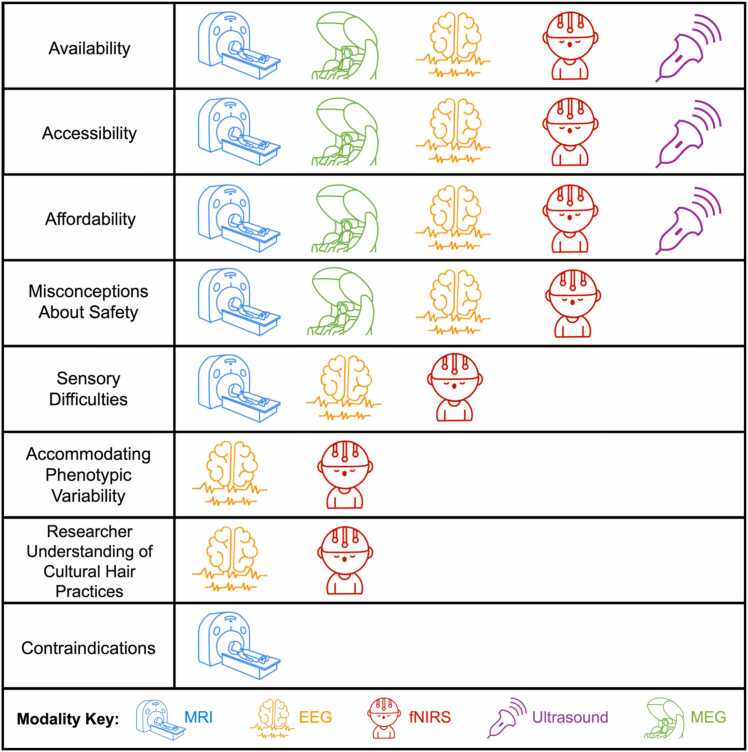
Identified challenges to improving representation in FIT research samples by modality.

Using a modality-level approach, we described current limitations (summarized in Fig. 2) and highlighted ongoing initiatives to improve representation in research samples. In the following discussion, we address several overarching directions for the FIT neuroimaging field to improve inclusivity and representation of those from the global majority and with marginalized identities. Table 2 includes additional resources that elaborate upon many of these directions.

### 3.1 Diversification of research teams

It is imperative to acknowledge the impact of representation of diverse communities among the staff engaged in data collection. For example, evidence suggests that having representative scientists on research teams may foster feelings of familiarity among participants which may increase trust in science (Flores et al., 2017, Tzovara et al., 2021, Wallace and Bartlett, 2013). Hiring and empowering research staff with identities/backgrounds matching those of research participants is key to effective recruitment and retention of participants and to enhance equity in FIT neuroimaging research overall (Adams et al., 2024, Kwasa et al., 2023, The BRIGHT Project team, 2023). Similarly, researchers with neurodiverse lived experience and/or parents of neurodiverse children can provide useful insight into the process of data collection (Serino et al., 2023).

### 3.2 Community advisory boards

Establishing community advisory boards, comprising community members from the population of interest and/or those with lived experiences, is a vital step in conducting community-based research (La Scala et al., 2023, Matthews et al., 2018, Newman et al., 2011). In doing so, FIT neuroimaging researchers can better learn about obstacles and barriers to participating in research from the viewpoint of community members and actively work together to address initiatives that are feasible. For example, community advisory boards may develop strategies to mitigate therapeutic misconceptions, test drive whether materials make sense in the cultural context, discuss appropriate literacy levels of materials, and develop policies for dissemination of results and/or incidental findings.

### 3.3 Culturally responsive and inclusive communication

Staff training in cultural responsiveness and respectful, transparent communication is key to building trust and rapport with participants, particularly with communities that have been historically exploited in research. Clear and culturally responsive communication about study procedures, as well as emphasizing participant agency and boundary-setting throughout the research process is vital. In addition, researchers should use language that is inclusive and respectful of diverse identities and family structures throughout the study (i.e., screeners, consents, instructions, questionnaires, publications). Researchers can opt for more general, inclusive language first and then follow participants’ lead/ask for preferences on terminology. Table 1 includes helpful resources for introducing neuroimaging modalities to families and children and Table 2 includes a list of helpful resources for more inclusive language. Additionally, research teams should include members who are fluent in the primary languages of their participant communities to ensure that all families have an equal opportunity to participate in the research. However, researchers must ensure that the level of fluency on their research team is adequate to provide a consistent and sufficient level of informed consent and explanation of procedures. Alternatively, researchers can make use of trained medical interpreters and translators.

### 3.4 The use of inclusive technologies

FIT neuroimaging equipment is not “one size fits all,” and additional attention to equipment may be necessary to ensure comfortable and successful data collection across phenotypes. Researchers should factor these considerations into their grant applications, and grant providers should also build expectations that these inclusive technologies are applied for and used to build equitable science. By integrating inclusive technologies into their research, researchers can ensure that their studies are accessible to all participants, ultimately leading to more inclusive and representative scientific outcomes.

### 3.5 High resource requirements for inclusive research

Substantial financial and other resource investments are required to recruit and retain diverse and marginalized populations in FIT neuroimaging studies. Unlike other areas of developmental research, most data collection for FIT neuroimaging cannot occur at participants' homes, posing logistical difficulties and child-care related barriers (Zgierska et al., 2024). Therefore, sufficient staff resources must be allocated to recruit and retain participants. Monetary resources are necessary to provide/reimburse reasonable lodging accommodation (e.g., in the case of nighttime visits), transportation, childcare, food/formula, and to appropriately compensate participants for their valuable time and effort. These logistical supports are crucial for successful data collection and ensuring that participation is accessible to all, regardless of socioeconomic status. This increased resource cost for representative FIT neuroimaging studies must be accounted for by grant providers and investigators in the grant process.

### 3.6 Researcher-developer-community collaborations to improve technology

It is essential to design and refine neuroimaging equipment that accommodates the diverse needs of all FIT populations. Successful innovations require feedback from researchers who understand the application and interpretation of technologies, engineers who design the technologies, and community members who can provide valuable insight from the participant point of view. These collaborations can lead to innovations that ensure no child is excluded due to current technological limitations.

### 3.7 Reporting on representative recruitment efforts and reasons behind sampling biases

Including key methodological information (i.e., recruitment and retention strategies, reasons for data exclusion, and demographic reporting) in reports is another way researchers can promote transparency and accountability in the representation of their FIT neuroimaging samples (Gard et al., 2024). Studies should explicitly detail recruitment and retention efforts, including the strategies utilized to reach diverse communities and the resources allocated to support participant engagement (Kwasa et al., 2023). Next, explicit disclosures and justifications for data exclusion (e.g., poor signal quality from suboptimal optode-scalp coupling) are necessary to promote accountability around exclusions and attrition that may be particularly salient to those from marginalized populations and culturally diverse communities (Kwasa et al., 2023). Additionally, researchers recommend reporting demographic information to promote transparency and accountability of samples represented in the research as well as ensure appropriate generalization (Girolamo et al., 2022; Girolamo et al., 2023, Kwasa et al., 2023, Morales et al., 2024). One excellent unified approach to collecting and reporting on culturally diverse demographic information in developmental research was introduced by Singh and colleagues (2023). By openly discussing these efforts, researchers can promote accountability, demonstrate their commitment to inclusivity and representation, thereby providing a roadmap for others in creating more equitable studies.

### 3.8 Increasing the global density of neuroimaging technologies

Expanding access to neuroimaging technologies worldwide is essential for promoting equitable science and enables more comprehensive and representative research. Establishing neuroimaging facilities in global majority countries and human capacity building (Cottler et al., 2015, Lloyd-Fox, 2023) in local communities will enable researchers there to independently and sustainably continue research, thereby ensuring that diverse populations continue to be represented in research. Considerations for human capacity building include developing hubs of expertise in content areas that make sense for the region/population, establishment of institutional programs and research infrastructure, as well as scaffolded mentorship and funding opportunities for trainees. For instance, RAD-AID – a nonprofit organization that brings radiology resources (both technology and training) to hospitals in low-resource regions of the world (Mollura et al., 2019) – has been actively working to reduce barriers to accessing lifesaving diagnostic tools, such as prenatal ultrasound, to parts of the world where it would not be possible to access such services otherwise. The global density of neuroimaging technologies can also be improved via global research collaborations between researchers who have experience with these technologies and researchers looking to implement particular modalities locally. Researchers interested in these types of collaborations should look to the TRUST Code, a framework for the ethical conduct of global research partnerships between countries with differential resources (TRUST, 2018). Examples of global research partnerships with human capacity building include the BRIGHT project in the Gambia leveraging fNIRS (Katus et al., 2019, The BRIGHT project team, 2019) and the UNITY project in many countries across the Global South using low-field MRI (Abate et al., 2024). We must also acknowledge the financial dimension of improving global neuroimaging density. Empirical evidence suggests that the amount of neuroimaging research published across a number of modalities discussed in the current article (i.e., MRI, MEG, EEG, fNIRS) is related to a country's economic resources, with increased resources being related to more neuroimaging research (Duncan and Rae, 2024). In this line of thought, technology developers and grant funders should also recognize that buying power varies significantly by region, meaning technology considered more affordable in high-resource areas (e.g., EEG) may be prohibitively expensive in low-resource settings. Stakeholders can look to other neuroimaging modalities, such as ultrasound, as a technique that remains highly cost-effective across economic contexts. To enhance accessibility, companies should consider adopting sliding scale pricing models tailored to regional economic disparities.

### 3.9 Conducting multisite, large-scale studies

Large-scale, multi-site studies are another approach to improving inclusivity in FIT neuroimaging research. The multi-site design ensures that research findings are increasingly nationally or globally representative, which is especially important in the development of standard and normative charts used by clinicians. For instance, the Fetal Growth Longitudinal Study (FGLS; (Villar et al., 2013) from the International Fetal and Newborn Growth Consortium for the 21st Century (INTERGROWTH-21st) has established international standards for fetal growth velocity using ultrasound measurements, aiding in global monitoring of fetal brain growth. Another recent example is the HEALthy Brain and Child Development (HBCD) Study in the United States, which is the largest multimodal multisite study of early neurodevelopment across 27 recruitment sites (Jordan et al., 2020, Volkow et al., 2024).

## 4 Conclusion

Taken together, FIT neuroimaging researchers have an ethical and scientific responsibility to improve the inclusion of diverse research samples, particularly increasing representation from countries within the global majority and from marginalized communities, to develop a more equitable, reliable, and generalizable construct of the developing brain. While there is still vital work to be done in each modality highlighted in this article, many researchers, developers, grant-funding agencies, and community members have already demonstrated their commitment to this goal through the promising initiatives summarized in this article. Continued efforts addressing the overarching directions that transcend individual modalities will further extend the momentum of these modality-level efforts and push the field forward.

## Funding

– J.L.Y.C is supported by the 10.13039/501100000268Biotechnology and Biological Sciences Research Council [grant number BB/T008709/1].
– C.B. acknowledges support from the Early Career Fellowship Leverhulme Trust (ECF-2021–174).
– S.V.T.R acknowledges funding from R00HD104923.
– M.S. acknowleges support from 10.13039/100000025NIMH K24 MH127381.
– M.K. acknowledges that this research was supported [in] part by the Intramural Research Program at 10.13039/100000002NIH. Annual Report Number: ZIA-MH002781.
– The Fetal, Infant, and Toddler Neuroimaging Group acknowledge support from the Eunice Kennedy Shriver National Institute of Child Health and Human Development (10.13039/100000071NICHD) R13 HD108938.

## Declaration of Competing Interest

The authors declare that they have no known competing financial interests or personal relationships that could have appeared to influence the work reported in this paper.

## Footnotes

2 FIT: Fetal, infant, and toddler; MRI: Magnetic resonance imaging; EEG: Electroencephalography; fNIRS: Functional near-infrared spectroscopy; MEG: Magnetoencephalography
